# Plasticity of maternal environment-dependent expression-QTLs of tomato seeds

**DOI:** 10.1007/s00122-023-04322-0

**Published:** 2023-02-22

**Authors:** Mark G. Sterken, Harm Nijveen, Martijn van Zanten, Jose M. Jiménez-Gómez, Nafiseh Geshnizjani, Leo A. J. Willems, Juriaan Rienstra, Henk W. M. Hilhorst, Wilco Ligterink, Basten L. Snoek

**Affiliations:** 1 Laboratory of Nematology, Wageningen University, 6708 PB Wageningen, The Netherlands; 2 Wageningen Seed Lab, Laboratory of Plant Physiology, Wageningen University, 6708 PB Wageningen, The Netherlands; 3 Laboratory of Bioinformatics, Wageningen University, 6708 PB Wageningen, The Netherlands; 4 Plant Stress Resilience, Institute of Environmental Biology, Utrecht University, 3584 CH Utrecht, The Netherlands; 5 Department of Plant Breeding and Genetics, Max Planck Institute for Plant Breeding Research, Cologne, Germany; 6 Institut Jean-Pierre Bourgin, INRAE, AgroParisTech, Université Paris-Saclay, 78000 Versailles, France; 7 Theoretical Biology and Bioinformatics, Institute of Biodynamics and Biocomplexity, Utrecht University, 3584 CH Utrecht, The Netherlands

## Abstract

Seeds are essential for plant reproduction, survival, and dispersal. Germination ability and successful establishment of young seedlings strongly depend on seed quality and on environmental factors such as nutrient availability. In tomato (*Solanum lycopersicum*) and many other species, seed quality and seedling establishment characteristics are determined by genetic variation, as well as the maternal environment in which the seeds develop and mature. The genetic contribution to variation in seed and seedling quality traits and environmental responsiveness can be estimated at transcriptome level in the dry seed by mapping genomic loci that affect gene expression (expression QTLs) in contrasting maternal environments. In this study, we applied RNA-sequencing to construct a linkage map and measure gene expression of seeds of a tomato recombinant inbred line (RIL) population derived from a cross between *S. lycopersicum* (cv. Moneymaker) and *S. pimpinellifolium* (G1.1554). The seeds matured on plants cultivated under different nutritional environments, i.e., on high phosphorus or low nitrogen. The obtained single-nucleotide polymorphisms (SNPs) were subsequently used to construct a genetic map. We show how the genetic landscape of plasticity in gene regulation in dry seeds is affected by the maternal nutrient environment. The combined information on natural genetic variation mediating (variation in) responsiveness to the environment may contribute to knowledge-based breeding programs aiming to develop crop cultivars that are resilient to stressful environments.

**Supplementary Information:**

The online version contains supplementary material available at 10.1007/s00122-023-04322-0.

## Introduction

Seeds are essential for reproduction and dispersal of plants and function as survival structures to overcome harsh environmental conditions unfavorable for plant growth. Well-timed development and ripening of seeds, to ensure optimal seed performance and the ability to germinate in a permissive environment, are therefore essential for plant fitness. Successful germination strongly depends on seed performance, which is affected by environmental conditions, such as temperature, water availability, light conditions, and the nutrient status that the maternal plant experienced (de Souza et al. 2020; Delouche and Baskin 1971; Delouche 1980; Donohue 2009; Rowse and Finch-Savage 2003). More specifically, seed performance/germination in species such as tomato and the model plant *Arabidopsis thaliana* is determined during seed development and maturation and depends on temperature (Demir et al. 2004; He et al. 2014; Schmuths et al. 2006), photoperiod (Munir et al. 2001; Pourrat and Jacques 1975), nutrient composition, and levels (Alboresi et al. 2005; Geshnizjani et al. 2019; He et al. 2014). Seed quality, germination, and seedling establishment traits also have strong genetic determinants, and (natural) genetic variation in quality traits, including Quantitative Trait Loci (QTLs), has been reported (Geshnizjani et al. 2019, 2020; He et al. 2014; Joosen et al. 2012; Khan et al. 2012; Serin et al. 2017).

Phosphate and nitrate are essential plant nutrients with profound effects on plant growth (Schachtman et al. 1998; Urbanczyk-Wochniak and Fernie 2005) and seed performance/germination traits (Alboresi et al. 2005; Geshnizjani et al. 2019, 2020; He et al. 2014). In Arabidopsis, it has been shown that seeds produced by plants fertilized with higher-than-normal levels of phosphate showed increased germination rates under stressful conditions (He et al. 2014). Nitrate is known to have a strong effect on seed germination and seed dormancy in multiple plant species (Duermeyer et al. 2018), with high concentrations of nitrate supplied to the mother plant leading to lower dormancy of the seeds (Alboresi et al. 2005). This is attributed to nitrogen effects on the gibberellin/abscisic acid (GA/ABA) balance in the seeds, with higher endogenous nitrate levels resulting in lower ABA levels in seeds and hence shallower dormancy (Matakiadis et al. 2009). In Arabidopsis, altered nitrate levels experienced by the mother plant also have a substantial effect on the levels of multiple metabolites and transcripts in the seeds, with a notable reduction in nitrogen metabolism-related metabolites and genes (He et al. 2016).

Tomato (*Solanum lycopersicum*) is one of the most important vegetable crops worldwide and is a model organism for research on fruit-bearing crops (Giovannoni 2001; Schauer et al. 2006; Tomato Genome 2012; Tomato Genome Sequencing et al. 2014). However, in the process of domestication, breeding selection and propagation, a substantial fraction of the genetic variation in the founder’s germplasms has been lost (Razifard et al. 2020; Tomato Genome 2012; Tomato Genome Sequencing et al. 2014). Moreover, due to a focus on fruit quality, resistance, and yield traits, other desirable traits that have not been directly selected for could have been lost over time in modern varieties (McCouch 2004; Razifard et al. 2020; Wang et al. 2020). In plants other than tomato, this includes several seed quality traits (Bauchet et al. 2014; Doebley et al. 2006; McCouch 2004; Razifard et al. 2020; Wang et al. 2020). Trait variation loss could be restored by including wild cultivars/ancestors of modern commercial tomato such as *Solanum pimpinellifolium.* Yet, although wild ancestors represent a rich source of genetic variation (although also underlying negative traits) in breeding programs and in studies on tomato (quantitative) genetics (Blanca et al. 2015; Lin et al. 2014; Pascual et al. 2016; Razali et al. 2018; Tomato Genome 2012; Yang et al. 2014). For instance, wild cultivars have been used in genetic screens and genome wide association studies (GWAS) to discover genomic loci and genes involved in variation in metabolic traits (Bauchet et al. 2017; Sauvage et al. 2014; Ye et al. 2019; Zhang et al. 2015; Zhao et al. 2019), insect resistance (Vosman et al. 2018), floral meristem identity (Bauchet et al. 2014), trichome formation (Chang et al. 2018), microbial rhizosphere composition (Oyserman et al. 2022), and fruit shape and size (Albert et al. 2016; Blanca et al. 2015; Razifard et al. 2020). In addition to GWAS, Recombinant Inbred Line (RIL) populations, derived from experimental crossing between *S. lycopersicum* and *S. pimpinellifolium,* are frequently used to uncover the effect of genetic variation on tomato traits (Capel et al. 2015, 2017; Celik et al. 2017; Kazmi et al. 2017; Viquez-Zamora et al. 2014; Voorrips et al. 2000; Zhang et al. 2018), including various seed quality traits (de Souza et al. 2016; Geshnizjani et al. 2018, 2019, 2020; Khan et al. 2012).

The introduction and improved feasibility of diverse omics techniques have accelerated studies into the molecular mechanisms underlying natural variation in tomato traits in the past two decades (Rothan et al. 2019). In particular, advances in transcriptomics techniques such as microarray analysis and later RNA-sequencing have proved useful in this context, by enabling, e.g., GWAS studies. Moreover, measuring gene expression in RILs has enabled expression-QTL (eQTL) analysis as a powerful tool to detect gene regulatory loci (Jansen and Nap 2001; Jimenez-Gomez et al. 2010; Kawakatsu et al. 2016; Keurentjes et al. 2007; Snoek et al. 2012; West et al. 2007). Combining the wealth of information obtained by mapping eQTLs enables (re)construction of regulatory networks underlying plant traits (Jimenez-Gomez et al. 2010; Keurentjes et al. 2007; Terpstra et al. 2010). In addition, comparison of eQTL profiles from multiple environments may aid our understanding of how genetic variation shapes the effects the environment has on the appearance of phenotypes (Hartanto et al. 2020; Nijveen et al. 2017; Snoek et al. 2012). In plant (Arabidopsis) and worm (*Caenorhabditis elegans*) model systems, it has been shown that especially *trans-*eQTLs are dynamic and can be highly specific for a certain environment (Cubillos et al. 2014; Hartanto et al. 2020; Nijveen et al. 2017; Snoek et al. 2017, 2012; Sterken et al. 2019; Vinuela et al. 2010).

Although seed quality and seedling establishment characteristics are determined by both genetic variation and the maternal environment in which the seeds develop and mature (Geshnizjani et al. 2019, 2020; He et al. 2014), it is currently unknown if the maternal environment causes a perturbated eQTL landscape in the progeny seeds and how the nutrient environment of the mother plant affects these landscapes. We therefore followed an RNA-seq approach and quantified natural variation in mRNA levels in the dry seeds of a tomato RIL population from a cross derived from *S. lycopersicum* (cv. Moneymaker) and *S. pimpinellifolium* (G1.1554) parents (Khan et al. 2012; Voorrips et al. 2000) that were cultivated either in a low nitrogen or a high phosphorus environment. In this work, we first present a high-density RNA-seq-derived genetic map of tomato, and subsequently, we demonstrate how the genetic landscape of gene regulation of tomato dry seeds is affected by the nutritional environment of the mother plant. Altogether, our detailed analysis of the genetic underpinning of plasticity in gene expression as responsiveness to the maternal environment, attributed to the progeny seeds, may contribute to knowledge-based breeding programs aiming to develop crop cultivars that are resilient to stressful environments, including production of high-quality seeds under sub-optimal environmental conditions.

## Results

### An RNA-seq-derived genetic map of tomato

We performed an RNA-sequencing experiment to uncover the interplay between genetic variation, the nutritional status of the maternal environment, and mRNA abundances in progeny tomato seeds. The used seeds were derived from 101 tomato RIL plants of a cross between *S. lycopersicum* (cv. Moneymaker; MM) and *S. pimpinellifolium* (G1.1554 or CGN14498; PI) (Kazmi et al. 2012; Voorrips et al. 2000) and their parental lines. All maternal plants were pre-cultivated on standard nutrient conditions and upon flowering transferred to either low nitrogen (LN; 52 RILs) or high phosphate (HP; 49 RILs) nutrition. The two RIL sets were non-overlapping (Geshnizjani et al. 2020).

**Fig. 1:**
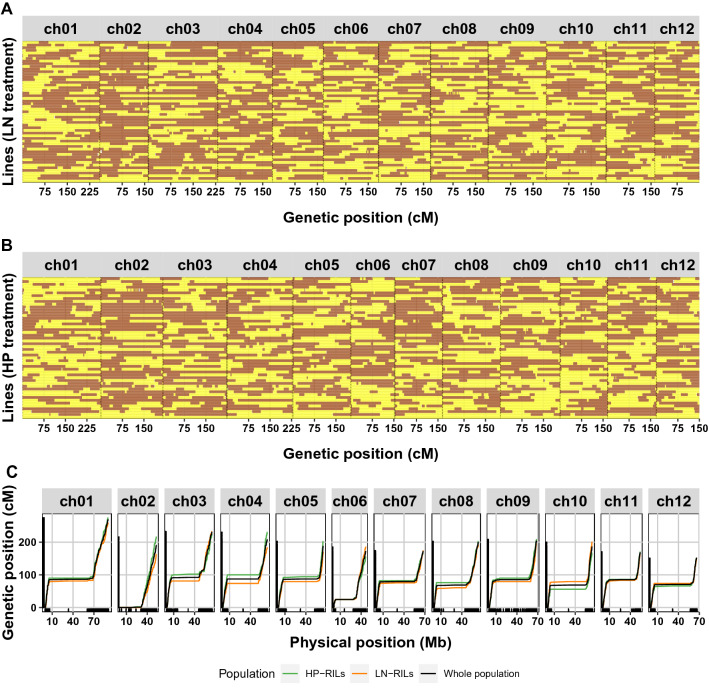
**a** Genetic map showing the genotypes of the RILs that were included in the low nitrogen (LN) treatment experiment. The map represents the most probable genotype per marker location. Yellow indicates MM; brown indicates PI. Position on the chromosome is indicated in centimorgans (cM). **b** As in (**a**), but for the RILs that were included in the high phosphorous (HP) treatment experiment. **c** Recombination events per chromosome for the whole population (black) and the LN-(orange) and HP-treated (green) sub-populations. The physical position is given on the *x*-axis in million bases (Mb); the genetic position is given on the y-axis in centimorgans (cM). Chromosome numbers are indicated above panels (color figure online)

In addition to estimating expression differences among individuals, RNA-seq reads allowed for the identification of single-nucleotide polymorphisms (SNPs) in transcribed genes of the parental lines and the RILs. These SNPs were subsequently used to construct genetic and physical maps of the RIL population, to facilitate QTL and eQTL mappings (Serin et al. 2017; Snoek et al. 2019). In total, we detected 43,188 consistent SNPs between the parental lines. These SNPs were subsequently used to reconstruct the genotypes (*i.e.,* determine the crossover locations) of the RILs (Fig. 1a, b). Across our RIL set, a balanced distribution of the parental alleles was observed genome-wide, with the notable exception of chromosome 2, which had a substantial higher frequency of PI alleles (Supplementary Fig. 1). Measured over all RILs, 2847 recombination (crossover) events were detected. As expected, the crossovers were found almost exclusively in euchromatic regions of the chromosomes, causing severe distortion between the physical and genetic maps, as described before (Demirci et al. 2017) (Fig. 1c). On average, two recombination events were detected per RIL per chromosome. Altogether, the population size and recombination events provided 4515 unique genetic markers and 4568 distinguishable genomic loci/bins suitable for mapping, improving the previously available map (Kazmi et al. 2012) (Supplementary Table 1). The detected loci had a size range from 60 to 1.7 Kb, with an average locus size of 180 Kb and a median of 11 Kb (Supplementary Table 2). Given the high local recombination frequency, relatively small loci were overrepresented toward the chromosome tips (Fig. 1c). Together, our dataset enables precise mapping of QTLs and eQTLs, especially toward the tips of the chromosomes but with poor resolution near the centromeres.

### The maternal nutrient environment affects mRNA abundances in seeds

**Fig. 2:**
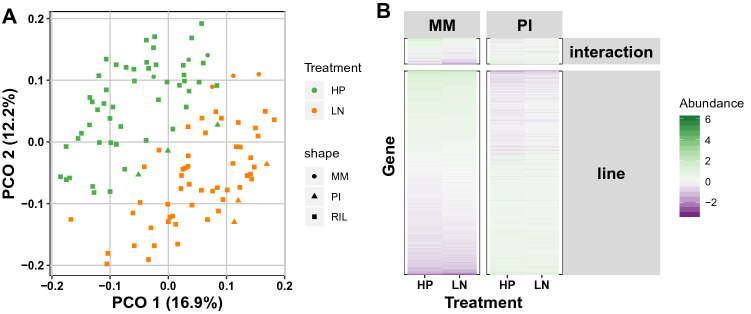
Nutrition status-related mRNA abundance differences. **a** The first two axes of a principal component analysis on the log_2_ ratio with the mean transcripts per million (TPM) values. The first axis (PCO1) explained 16.9% of the variance in the data, the second 12.2%. Square symbols represent individual RILs; Moneymaker (MM) parental samples are represented by dots and *S. pimpinellifolium* (PI) parental samples by triangles. The colors indicate high phosphorous (HP; green) or low nitrogen (LN; orange) treatments. **b** Differentially abundant mRNAs in the two parental lines not affected by treatment (2976 genes, line) and affected by treatment (382 genes, interaction). Lower abundance is shown in purple and higher in green. The genes can be found in Supplementary Table 5 (color figure online)

Next, we compared mRNA abundances in all HP-treated lines (RILs and parental lines) with the mRNA abundances in LN-treated lines, to identify genes contributing to differences between the two environments. Principal Component Analysis (PCA) demonstrated the presence of a substantial effect of the maternal nutrient environment on transcript levels in seeds (Fig. 2a). A linear model was used to identify which mRNAs were differentially expressed between the two maternal environments. A multiple testing correction was applied, and differential expression of 2871 mRNAs (out of 14,772 detected mRNAs) was found (Bonferroni corrected *p*-value < 0.05) to depend on the nutritional conditions the mother plant experienced during the seed maturation phase (i.e., LN or HP) (Supplementary Table 3). Of these 2871 mRNAs, 922 were more abundant in seeds developed and ripened in HP conditions compared to LN, and 1949 mRNAs were significantly more abundant in LN conditions compared to HP. The mRNAs of genes that were more abundant after LN treatment were among others enriched for Gene Ontology (GO) terms: ‘chloroplast,’ ‘ATP binding,’ ‘proteasome,’ and ‘nitrate transport’ (Supplementary table 4). mRNAs that were more abundant in seeds grown in HP conditions were enriched for the GO terms: ‘cellular response to hypoxia,’ ‘pectin esterase activity,’ and ‘glucosinolate metabolic process’ (Supplementary Table 4).

We also inquired the differences of the mRNA abundances between the MM and PI parental lines, within and between treatments. To this end, we again employed a linear model, but were less stringent in the statistical thresholds (as there were no confounding effects). We found 2976 mRNAs differentially expressed between the two parental lines regardless of treatment and 382 mRNAs that were differentially expressed between the lines due to treatment (linear model, FDR ≤ 0.05; Fig. 2b and Supplementary table 5). GO enrichment indicated that the 1240 mRNAs more abundant in MM compared to PI were, among other categories, enriched, for ‘transcription factor activity,’ ‘oxidation–reduction,’ ‘protein -binding,’ ‘-phosphorylation,’ ‘-ubiquitination,’ ‘chloroplast,’ ‘circadian rhythm,’ and ‘metal ion binding’ (Supplementary Table 6a). The 1736 mRNAs that were more abundant in PI compared to MM were, among other categories, enriched for ‘cytosol,’ ‘chloroplast,’ ‘nucleus,’ ‘mitochondrion,’ ‘cytoplasm,’ ‘ribosome,’ ‘translation,’ ‘nucleolus,’ ‘endoplasmic reticulum,’ ‘oxidation–reduction,’ ‘vacuole,’ and ‘copper ion binding’ (Supplementary table 6a). The 382 genes showing a significant interaction effect between the parental background and maternal environment showed an enrichment for the GO terms ‘oxidation–reduction,’ ‘extracellular region,’ ‘transcript regulation,’ ‘iron ion binding,’ and ‘response to gibberellin’ (Supplementary table 6b). Of note, the ‘oxidation–reduction process’ and ‘transcript regulation’ GO terms are enriched in the upregulated genes of both MM and PI, which is not surprising since both GO terms are quite general and each represents many genes. These results show that the nutrition status of the mother plant (environment; E) as well as genotype (G), and the interaction between the two (G × E), modulates mRNA abundances in dry seeds of tomato.

### Heritability and transgression in mRNA abundances

**Fig. 3:**
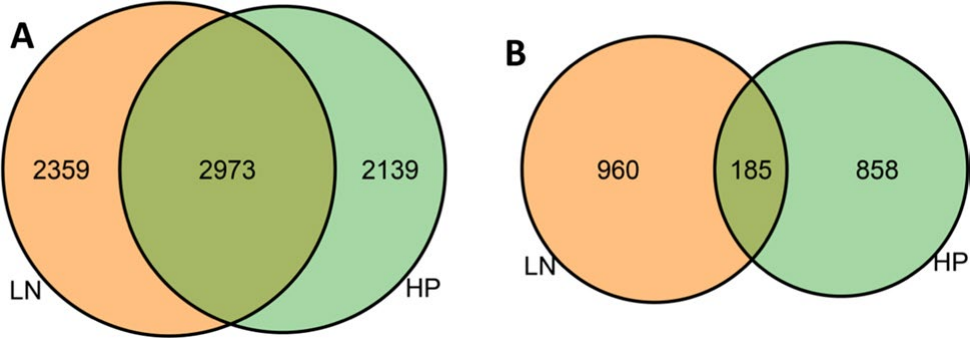
Venn diagrams showing the overlap and differences of **a** genes with significant heritability and **b** genes exhibiting significant transgression, of mRNA abundance levels between LN (orange) and HP (green; FDR < 0.05) (color figure online)

To estimate the contribution of genetic variation to differences in mRNA abundance between the genetic backgrounds (plant lines) and treatments (nutrient status), we calculated the Broad-Sense Heritability (BSH). It should be noted that the method used in general gives an upper-bound estimation of the BSH (Brem and Kruglyak 2005; Keurentjes et al. 2007; Rockman et al. 2010; Snoek et al. 2012; Sterken et al. 2019). In addition, replicated measurements in the parental lines were used to estimate non-genetic variance. We found 5112 genes in HP and 5332 genes in LN that showed significant heritability for mRNA abundance, of which 2973 genes overlapped (39.8%; permutation, FDR < 0.05; Fig. 3a; Supplementary Table 7a). Subsequently, we checked if genes with significant heritable contribution to mRNA abundance differences were predominantly affected by the maternal nutrient environment. However, we did not find such an enrichment for any of the overlapping groups of genes (hypergeometric test, *p* > 0.01; Supplementary Fig. 2a). We thus conclude that, overall, the number of genes with significant heritability for mRNA abundance was not specifically responsive to the maternal nutrient treatments. The genes with heritable mRNA abundance in HP alone were enriched for the GO terms: ‘translation,’ ‘ribosome,’ ‘mitochondrion,’ and more (Supplementary Table 7b). Those that showed significant heritability only in LN were enriched for the GO terms: ‘ABA metabolic process,’ and others (Supplementary table 7b). The genes that showed significant heritability in both environments were enriched for various GO terms: ‘oxidation–reduction process,’ ‘ribosome/translation,’ ‘nucleolus,’ ‘cell wall,’ ‘heme binding,’ ‘ion binding,’ and ‘vacuole’ (Supplementary Table 7b).

We next assessed the complexity of the genetic regulation underlying mRNA abundance differences. To this end, the transgression was calculated, i.e., trait values in RILs that extent beyond the parental means. We found significant transgression in mRNA abundance (trait) levels for 1043 genes in the maternal HP treatment and 1145 genes in the maternal LN treatment (permutation, FDR < 0.05; Supplementary Table 8a). This suggests a polygenic genetic architecture for mRNA abundance. Of these, the mRNA abundances of 185 genes showed significant transgression beyond the parental means in both treatments (Fig. 3b). Also, here, we tested for significant overlap with treatment-related genes. Yet, with 18% response to treatment of the transgressive mRNAs, there was no significant enrichment for transgressive mRNA abundances with treatment-related differences (hypergeometric test, *p* > 0.01; Supplementary Fig. 2b). So, alike heritability, transgression is apparently not linked to a reduction of nitrogen or increase in phosphorus content in the maternal growth environment. Moreover, compared to genes showing significant heritability, many fewer GO terms were enriched in the genes showing transgression, and those GO terms that were enriched generally had a lower level of significance. For genes showing transgression in HP alone, the GO terms ‘cell periphery,’ ‘positive gravitropism,’ ‘cysteine biosynthetic process,’ ‘symporter activity,’ and ‘response to heat’ were enriched, whereas for genes only showing transgression in LN the GO-terms ‘beta-glucosidase activity,’ ‘preprophase band,’ and ‘phragmoplast’ were enriched. The GO term ‘DNA-binding transcription factor activity’ was enriched in genes showing transgression in both environments (Supplementary Table 8b).

### The maternal nutrient environment produces specific eQTL landscapes

**Fig. 4:**
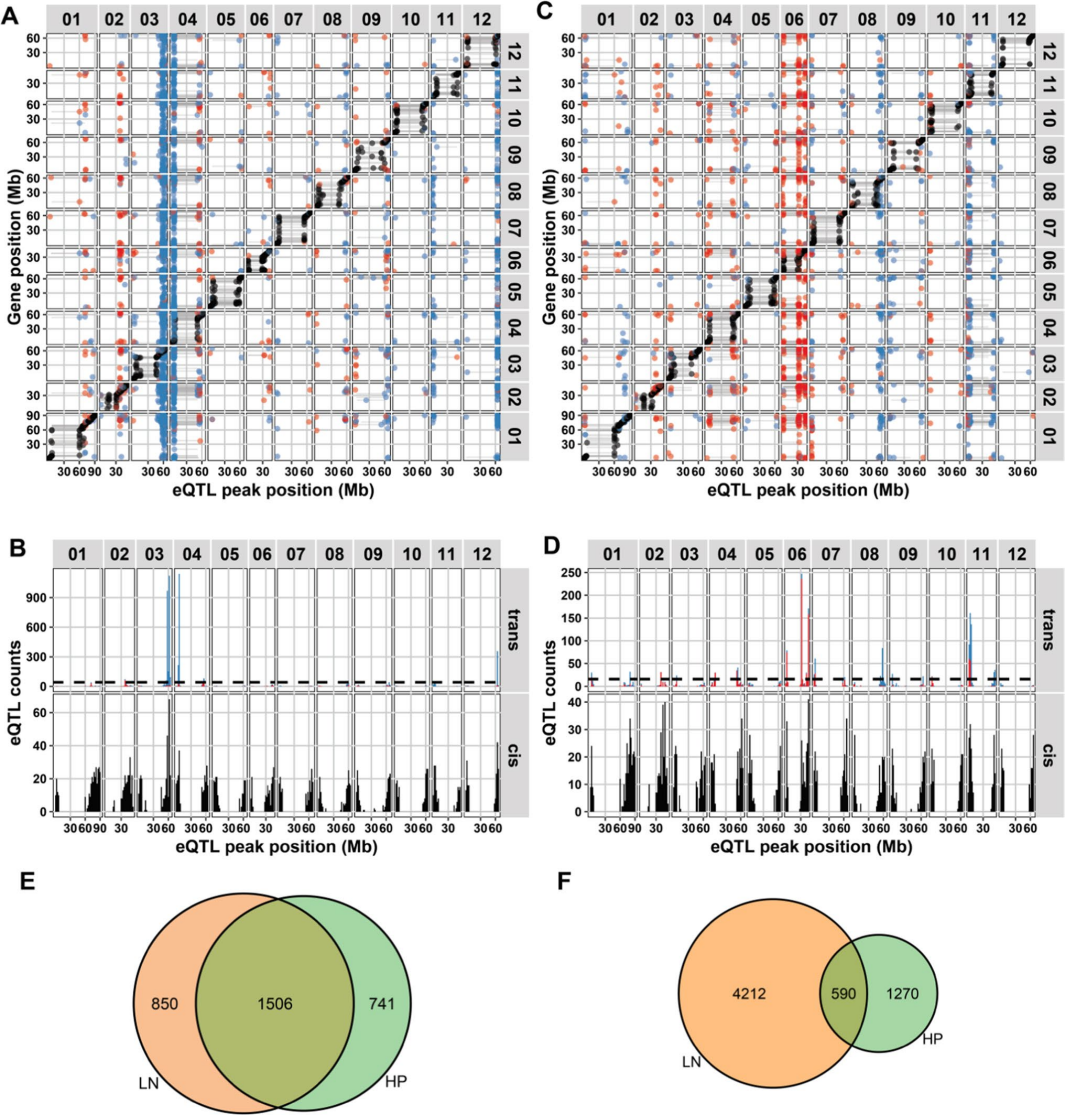
Characteristics of the detected eQTL landscapes in tomato dry seeds in **a,** **b** LN and **c,** **d** HP environments. **a, c** *Cis–trans* plots of eQTLs mapped (− log_10_(*p*) > 3.9). The positions of the eQTL peaks are plotted on the *x*-axis and the positions of the corresponding genes on the y-axis. Chromosomes are indicated on the top and right in the gray labels. Colors indicate *cis*-eQTL (black), eQTL associated with higher mRNA abundance due to the MM allele (blue) or with higher abundance by the PI allele (red). **b**, **d** Histograms showing the distribution of the *cis*- and *trans*-eQTL over the chromosomes, arranged by eQTL peak location counted per 2 million bases (Mb) bins. The dashed lines in the *trans*-eQTL panels indicate the threshold for calling a *trans*-band (Poisson distribution, *p* < 0.0001). **e** The overlap of *cis*-eQTL in the two treatments and **f** the overlap of *trans*-eQTL in the two maternal environments (color figure online)

Altogether, our analyses revealed both a considerable effect of the maternal nutrient environment (HP versus LN) and a significant influence of genetic variation in the RIL panel (heritability) on the detected mRNA abundance levels. By combining our constructed SNP genetic map (Fig. 1a, Supplementary table 1) with the obtained mRNA abundance dataset (Fig. 2), we were able to identify eQTLs that potentially contribute to the variation in mRNA abundance (Fig. 4a–f). In other words, the identified eQTL have a high chance of harboring polymorphic regulatory factors (e.g., genes or other genetic elements) for mRNA abundance, prospectively explaining variation in the seed and germination trait phenotypes observed.

We detected a maternal environment-specific *trans*-eQTL landscape, as the distribution of the position of the *trans*-eQTLs was very different between the two environments. For the HP environment, 4281 eQTLs for 3833 genes were identified, of which 2247 were *cis*-eQTLs and 2034 were *trans*-eQTLs. For the LN environment, 7487 eQTLs were detected for 6815 genes, of which 2356 were *cis*-eQTLs and 5131 were *trans*-eQTLs (FDR < 0.05; − log_10_(*p*) > 3.9; Fig. 4a–d; Supplementary Table 9; Supplementary Table 10). The confidence intervals of the eQTL locations were mostly dependent on the chromosomal area where the QTL was mapped, with small intervals at the chromosomal arms and large intervals nearer the centromeres (Supplementary Table 9). A significant overlap between *cis*-eQTLs of the two environments was noted (Fig. 4e; 1506 overlapping *cis*-eQTLs; 48.6%; hypergeometric test, *p* < 1*10^–16^). On the contrary, the *trans*-eQTLs were mainly specific for each tested maternal environment (Fig. 4f; 590 overlapping *trans*-eQTLs; 9.7%; hypergeometric test, *p* = 1.0). However, both *cis-* and *trans*-eQTLs were not enriched for genes with differentially abundant mRNA levels based on the maternal environment (hypergeometric test, *p* > 0.01; Supplementary Fig. 3a and b). Together with the significant transgression (Supplementary Table 8a) and considerable heritability of mRNA abundances (Supplementary Table 7a; Fig. 3a), this indicates that *trans*-eQTLs represent a genotype-specific interaction with the maternal nutrient environment. Many different GO terms were found to be enriched in the genes with environment-specific eQTLs. For an overview, see Supplementary table 11.

The majority of the *trans*-eQTLs clustered in maternal nutrient environment-specific eQTL hotspots or *trans*-bands (Fig. 4a, c). Hence, these genomic regions harbor the main loci underlying the genetic variation in environment-specific gene expression regulation in our dataset. A total of 13 *trans*-bands (9 in the HP treatment and 4 in the LN treatment; see Methods for the *trans*-band criteria) were identified, which account for 1206 of the *trans-*eQTLs in the HP treatment (59.3% of HP total) and 4181 of the *trans*-eQTLs in the LN treatment (81.5% of LN total; Table 1).

**Table 1:** Overview of detected *trans*-band (TB) eQTLs. Indicated are given ID’s, location on the physical genome (map position in Mb), number of eQTLs underlying the *trans*-band (+sign: MM > PI; −sign PI > MM), GO terms enriched in the eQTLs underlying the *trans*-band in either MM or PI and co-location with known phenotypic QTLs for germination (Geshnizjani et al. 2020; Khan et al. 2012); +sign: MM > PI; −sign PI > MM)

TB ID	Position	eQTLs	GO enrichment (+ MM higher)	GO enrichment (−PI higher)	Germination QTL Refs: (Geshnizjani et al. 2020)
LN_TB1	ch03: 56–64 Mb	2369 (2330+; 39−)	Translation; ribosome; nucleolus; RNA binding; mitochondrion; cell wall; and more	None	Th-I [57.5 Mb] (LN−)
LN_TB2	ch04:4–8 Mb	1348 (1311+; 37−)	Telomere; nucleus; protein binding; ubiquitin; and more	None	Gmax water [3.14 Mb] (LN+); T10 water [3.14 Mb] (LN+); T10 NaCl [3.14 Mb], mann [4.94 Mb], HT [3.14 Mb] (LN+); T50 water [3.14 Mb], mann [4.94 Mb], (LN+); AUC water [3.14 Mb], mann [3.14 Mb] (LN+)
LN_TB3	ch04:54–56 Mb	99 (45+; 54−)	None	Secretory vesicle; and more	Gmax NaCl [55.0 Mb], mann [58.2 Mb] (LN+); SW [50.2 Mb] (LN+)
LN_TB4	ch12:62–66 Mb	365 (348+; 17−)	Golgi; endosome; glycosylation; ER; and more	None	None in LN
HP_TB1	ch01:2–4 Mb	41 (16+; 25−)	None	None	None in HP
HP_TB2	ch04:6–8 Mb	38 (7+; 31−)	None	Heme binding; oxidoreductase; iron ion binding	Gmax mann [3.14 Mb], HT[3.14 Mb] (HP+); Th-I [1.90 Mb] (HP+)
HP_TB3	ch06:2–4 Mb	96 (5+; 91−)	None	None	U8416 NaCl [9.03 Mb], HT[9.03 Mb] (HP+)
HP_TB4	ch06:32–34 Mb	254 (12+; 242−)	None	RNA processing	Th-T [33.7 Mb] (HP+)
HP_TB5	ch06:44–48 Mb	182 (13+; 169−)	None	Tricarboxylic acid cycle; plastid; vacuolar membrane; cell wall	T10 NaCl [43.8 Mb], mann [43.8 Mb], HT [43.6b] (HP+); T50 NaCl [43.8 Mb], HAT [43.8 Mb] (HP+); AUC NaCl [43.8 Mb] (HP+)
HP_TB6	ch07:0–2 Mb	77 (32+; 45−)	None	None	None in HP
HP_TB7	ch08:58–60 Mb	83 (80+; 3−)	Vacuole; oxidoreductase; golgi	None	Th-D [59.6 Mb] (HP+)
HP_TB8	ch11:0–6 Mb	371 (249+; 122−)	chromosome, centromeric region; ubiquitin conjugating enzyme activity	Transferase activity; hydrolase activity; response to heat	None
HP_TB9	ch11:52–54 Mb	64 (63+; 1−)	Ribosome; nucleolus; translation; and more	None	T10 mann [48.3 Mb] (HP-)

Thus, *trans*-bands are a major explanatory factor for *trans*-eQTLs. In other words, a relatively large proportion of *trans*-eQTLs are caused by a few pleiotropic major effect loci. Remarkably, the MM allele had a positive effect on mRNA abundance for the majority of the eQTLs of the *trans*-bands in the LN soil environment, whereas this was not so prevalent in the HP environment (Table 1; Fig. 4a, c). Most of these *trans*-bands showed enrichment for specific GO terms, such as ‘translation’ and ‘specific cellular organelles' for LN and ‘oxidoreductase’ and ‘vacuole’ for HP (Table 1, Supplementary Table 12). Moreover, many of the *trans*-bands co-locate with known QTLs for germination and seed traits [Table 1 (Geshnizjani et al. 2020; Khan et al. 2012)]. These eQTLs can therefore contribute to uncovering the molecular genetic mechanisms underlying the germination and seed trait QTLs.

## Discussion

Our RNA-sequencing data obtained from a Tomato RIL population (*S. lycopersicum* (cv. Moneymaker; MM) × *S. pimpinellifolium* (G1.1554; PI)) (Kazmi et al. 2012; Voorrips et al. 2000) allowed for the construction of a genetic map, describing the genotypes using 4515 SNP markers. This is over five times more than previously reported in Kazmi et al. (2012), which used 865 markers. However, intrinsic to RNA-seq data, only SNPs present in the coding parts of the genes (mRNA’s) could be used. Therefore, determining the exact locus where recombination took place would need additional genome sequencing as described in (Demirci et al. 2017). Furthermore, recombination events were mostly limited to the chromosome arms, leading to a skew in mapping resolution, with more narrow QTLs on the arms when compared to those located more toward the centromere regions.

By measuring transcript levels (i.e., mRNA abundances) in the seeds of a tomato RIL population that had matured in different maternal nutrient environments, we show that the maternal environment affects both regulation and the genetic architecture of gene expression in progeny seeds. By design of the experiment which focusses on the role of genotypic variation within an environment, we can only observe the differences between the two (extreme) environments. However, this comparison would benefit from the contrasts between normal nutrient conditions and these more extreme environments. An RNAseq experiment on seeds coming from standard nitrogen/phosphorous conditions would surely benefit this analysis and might help identification of gene expression or even eQTL more specific to high nutrient environments.

The genetic analysis revealed that especially *trans* eQTLs proved environment specific, which is comparable to other species (Albert et al. 2018; Cubillos et al. 2014; Hartanto et al. 2020; Li et al. 2006; Nijveen et al. 2017; Snoek et al. 2017, 2020, 2012; Vinuela et al. 2010). We found 3833 genes (~ 26% of all detected expressed genes in the RILs), with an eQTL in HP and 6815 genes (~ 46% of all expressed genes in the RILs) with an eQTL in LN. This is comparable to the number detected by Ranjan et al. 2016 (Ranjan et al. 2016), who used the upper part of five-day-old hypocotyls of introgression lines (ILs), developed from the wild desert-adapted species *Solanum pennellii* and domesticated *Solanum lycopersicum* cv. M82 (Eshed and Zamir 1995), and found 5300 genes (~ 25% of total expressed genes) to have an eQTL, with roughly half in *cis* and half in *trans*. We also found this close to 50/50 ratio in the HP condition, whereas in the LN condition the ratio of *cis/trans* eQTLs was increased to 30/70. Research in yeast indicated that the detection of *trans*-acting eQTLs is more strongly affected by the power of the study than detection of *cis*-acting eQTLs (Albert et al. 2018). So, it is likely that in our study we would have even more *trans*-eQTLs relative to *cis-*eQTLs.

By comparing two different maternal environments in a population originating from two different genetic backgrounds, many different maternal environment-specific eQTLs were detected. This underlines the interplay between genetics and nutrient environment in our study. However, upon enrichment analysis of genes regulated by the environment-specific *trans*-eQTL hotspots, no obvious terms linking to the HP and LN environments were uncovered. Still, this information might be relevant for uncovering molecular mechanisms underlying the traits previously identified to co-locate with these *trans*-eQTL hotspots (Geshnizjani et al. 2020; Kazmi et al. 2012). Yet, we expect much of the variation caused by this interplay will be uncovered in future studies increasing numbers of different timepoints, environments, and genotypes. It should be noted that, considering the relatively limited amount of RILs (49 RILs in the HP treatment and 52 RILs in the LN treatment in this study), eQTL mapping power can be likely improved by measuring more RIL genotypes.

Mapping eQTL can help in the identification of causal genes underlying phenotypic QTL. In our study, the resolution for identifying causal genes is mostly limited by the recombination frequency in the population used, which is limited in RILs with on-average two crossovers per chromosome. Next to recombination events, identification of causal polymorphic genes in this and other eQTL studies can be assisted by using prior knowledge (Hartanto et al. 2022), and more detailed data on the number and type of polymorphisms between tomato lines, such as frameshifts (Kevei et al. 2015) and copy number variations (Razali et al. 2018). Moreover, combining eQTLs with QTLs obtained using phenotypic trait data (Geshnizjani et al. 2019, 2020; Khan et al. 2012), as well as other molecular data such as proteomics and/or metabolomics (Kazmi et al. 2017), will contribute to obtaining mechanistic insight on how genotypic variation leads to phenotypic variation between individuals at a systemic level. Furthermore, these eQTLs could be used as a lead in studies with a larger source of wild genotypes and combined with GWAS (Bauchet et al. 2017; Chang et al. 2018; Mata-Nicolas et al. 2020; Ye et al. 2019; Zhang et al. 2015; Zhao et al. 2019), to pinpoint causal polymorphisms underlying variation at both the molecular and phenotypic levels.

## Methods

### Plant lines, growth conditions, and nutrient treatments

The mother plants (maternal conditions) were cultivated as described in Kazmi et al. (2012) and Geshnizjani et al. (2020) (Khan et al. 2012), in the greenhouse at Wageningen University, the Netherlands. In short, the parental lines *Solanum lycopersicum* cv. Money maker (MM) and *Solanum pimpinellifolium* accession CGN14498 (PI) as well as the derived recombinant inbred lines (RILs; (Voorrips et al. 2000); Supplementary Table 1) were grown on rockwool under standard nutrient conditions (14 mM nitrate and 1 mM phosphate) with a 16-h light (25 °C) and 8-h darkness (15 °C) photoperiod. From the moment the first flower opened, the plants were fertilized with the specific nutrient solutions, low nitrate (2.4 mM Nitrate, 1 mM Phosphate), and high phosphate (14 mM Nitrate, 5 mM Phosphate) in two biological replicates per environment. The seeds were collected from healthy and fully ripened fruits, and the pulp still attached to the seeds was removed with 1% hydrochloric acid (HCl) and a mesh sieve (for 30 min). Water was used to remove the remaining HCl and pulp. For disinfection, seeds were treated with trisodium phosphate (Na_3_PO_4_⋅12H_2_O) for 15 min. Subsequently, seeds were dried at 20 °C for 3 days on a clean filter paper in ambient conditions. The seeds were then stored in paper bags at room temperature.

### RNA-isolation, library prep, and RNA-seq

We used 10 mg grinded powder derived from 30 whole, dry, brushed, after-ripened seeds (12 months after harvest) of parental lines, and the RILs grown under different nutrient environments in a generalized genetical genomics design (Li et al. 2008, 2009b) to extract total RNA. For the HP treatment, 3 replicates for the parental lines and 49 unique RILs were sequenced (one replicate per RIL). For the LN treatment, 3 replicates for the parental lines and 52 unique RILs were sequenced (one replicate per RIL) (Supplementary Table 13). RNA was isolated using the NucleoSpin RNA plant isolation kit (Macherey–Nagel 740,949) with on-column DNA digestion and adding Plant RNA isolation Aid (Life technologies) according to the manufacturer’s protocol and instructions. Strand-specific RNA-seq libraries were prepared from each RNA sample using the TruSeq RNA kit from Illumina according to manufacturer’s instructions. Poly-A-selected mRNA was sequenced using the Illumina HiSeq2500 sequencer, producing strand-specific single-end reads of 100 nucleotides. Raw sequence reads can be found in the Sequence Read Archive (SRA; www.ncbi.nlm.nih.gov/sra) under ID: PRJNA704909.

### Alignment and SNP calling

Reads were trimmed using Trimmomatic (version 0.33, (Bolger et al. 2014) to remove low-quality nucleotides. Trimmed reads were subsequently mapped to the Tomato SL4.0 reference genome with the ITAG4.0 annotation (Hosmani et al.^2020^) using the HISAT2 software (version 2.1.0 (Kim et al. 2015) with the –dta-cufflinks option. The resulting SAM alignment files were sorted and indexed using samtools version1.9 (Li et al. 2009a). SNPs were called using bcftools mpileup with a minimum read depth of 3.

### Generation of a physical and genetic map from RNA-seq data

The physical map used for mapping the eQTLs was made from the RNA-seq data following the protocol described in Serin et al. (2017) and Snoek et al. (2019). With the following modifications: SNPs were filtered for those that were consistently found in all replicates of the parental lines and observed in all RILs. Then, the genotype per RIL was determined per sliding bin of 100 SNPs where the mean position of those SNPs was taken as the physical position of the obtained marker.

The genetic map was constructed by converting the genotype probabilities to the most likely genotype and subsequently determining the number of recombinations between subsequent markers. The chance of recombination was used to generate a centimorgan (cM) map.

### Quantification of RNAseq

Before mRNA abundance analysis, between 12 and 31 M reads per sample were mapped to the SL4.0 genome with ITAG4.0 annotation (Hosmani et al. 2020) using HISAT2 as described above. The mRNA abundance was quantified to counts using Stringtie (Pertea et al. 2015) with the options -e, -B, and -G. In R, the counts were used to calculate transcripts per million (TPM). The TPM values were log_2_-transformed by

$${\mathrm{TPM}}_{\mathrm{log}}={\mathrm{log}}_{2}(\mathrm{TPM}+1)$$

Additionally, to use for statistics, also a ratio with the average was calculated, by

$${\mathrm{TPM}}_{\mathrm{rat},i,j}={\mathrm{log}}_{2}\left(\frac{{\mathrm{TPM}}_{i,j}}{{\overline{\mathrm{TPM}} }_{j}}\right)$$

where the log_2_ was calculated for each transcript *i* of sample *j* by dividing over the average value for that transcript $\overline{\mathrm{TPM} }$ over all samples *j*. After transformation, the transcripts were filtered for TPM_log_ > 0 and detection in all samples.

### mRNA abundance analysis and QTL analyses

The analyses reported below were conducted in “R” (version 3.5.3, × 64) (R-Core-Team 2017) with custom written scripts, accessible via https://git.wur.nl/published_papers/sterken_tomato-eqtl_2021. For analysis, the dplyr and tidyr packages were used for data organization (Wickham et al. 2018; Wickham 2018), and plots were generated using ggplot2 (Wickham 2009).

### Treatment-related mRNA abundance differences

The principal component analysis comparing the mRNA abundances was done on the *TPM*_*rat*_-transformed data, using the *prcomp* function in “R.” The mRNA abundance differences between treatments were tested between the LN and HP treatments using the linear model

$${\mathrm{TMP}}_{\mathrm{log},i}={T}_{i}+{e}_{i}$$

where TPM_log*,i*_ is the abundance level of transcript *i* (one of 14,772 transcripts) in RIL *j* (*n* = 55 for the HP treatment and *n* = 58 for the LN treatment; these include the RILs and the parental replicates per condition), *T* is the treatment (HP or LN), and *e* is the error term. To reduce the chance of detecting differences due to genetic variation, a strict multiple testing correction was applied (Bonferroni) using *p. adjust*. The threshold for significance was − log_10_(*p*) > 5.47 (FDR = 0.05).

To determine the effect of treatment on the differences in mRNA abundance between the parental lines, we ran a linear model explaining the differences due to treatment and line effects on the MM and PI parental data. The model used was

$${\mathrm{TMP}}_{\mathrm{log},i,j}={T}_{i,j}+{L}_{i,j}+{T}_{i,j}\times {L}_{i,j}+{e}_{i,j}$$

where TPM_log*,i,j*_ is the abundance level of transcript *i* (one of 14,772 transcripts) in parental replicate *j* (*n* = 3 for both treatments for MM and PI), *T* is the treatment (HP or LN), *L* is the line (MM or PI), and *e* is the error term. Values were corrected for multiple testing using *p. adjust* following the Benjamini–Hochberg algorithm. The thresholds for FDR = 0.05 were: − log_10_(*p*) = 1.71 for line, − log_10_(*p*) = 2.08 for treatment, and − log_10_(*p*) = 2.89 for the interaction between line and treatment. We took the most stringent *p* value, − log_10_(*p*) = 2.89 as threshold to determine significance.

### Transgression

Transgression was calculated by counting the number of lines with expression levels beyond three standard deviations from the mean of the parental lines (as in Brem and Kruglyak 2005); *µ* ± 3*σ. This was done for both treatments separately. The lower boundary was established by the parental line with the lowest mean, and the upper boundary was established by the parental line with the highest mean. The standard deviation used to determine transgression (*σ*) was calculated as the pooled standard deviation of the two parental lines (*n* = 3 for both).

Significance of the transgression was calculated by permutation. The expression values were randomized over the line designations and the same test as above was conducted. This was repeated 1000 times for each transcript, so the obtained values could be used as the by-chance distribution. The 50th highest value was used as the false discovery rate (FDR) = 0.05 threshold.

### Heritability

The heritability was calculated by estimating the genotypic variance in the RILs and the remaining variance (e.g., measurement error) in the parental lines (as in Keurentjes et al. (2007)). This was done for both treatments separately, by

$${H}_{\mathrm{RIL}}^{2}=\frac{{V}_{\mathrm{RIL}}-{V}_{\mathrm{e}}}{{V}_{\mathrm{RIL}}}$$

where *V*_RIL_ is the variance within the RIL population and *V*_e_ is the pooled variance of both parental lines.

To establish whether the heritability was significant and not outlier driven, we applied a permutation approach [as in Vinuela et al. (2012)]. The trait values were randomized over the line designations and the heritability calculation were repeated. This was done 1000 times for each transcript to generate a by-chance distribution. The 50th highest value was used as the FDR = 0.05 threshold.

### eQTL mapping

For eQTL mapping a single marker model was used and was applied separately for both treatments (as in (Snoek et al. 2017; Sterken et al. 2017)). QTLs were mapped using the model

$${\mathrm{TPM}}_{\mathrm{log},i,j}={x}_{j}+{e}_{j}$$

where TPM_log*,i,j*_ is the expression level of transcript *i* (one of 14,772 transcripts) in RIL *j* (*n* = 49 for the HP treatment and *n* = 52 for the LN treatment). The expression levels were explained over the genotype on marker location *x* (*x* = 1, 2,…, 4515) of RIL *j*.

To determine the reliability of the detected QTLs and correct for multiple testing, a permutation approach was used. As in the other permutations, the expression levels were randomly distributed over the lines and this randomized set was mapped again according to the procedure described above, which was repeated 10 times. To determine the FDR, we applied a correction for multiple testing under dependency (Benjamini and Yekutieli 2001)

$$\frac{\mathrm{FDS}}{\mathrm{RDS}}\le \frac{{m}_{0}}{m}\times q\times \mathrm{log}(m)$$

where FDS (false discovery) is the number of eQTLs detected in the permutation and the RDS (real discovery) is the number of eQTLs detected in the QTL mapping at a specific significance level. The number of true null hypotheses tested (*m*_0_) was 14,772*-RDS*, where the number of hypotheses tested (*m*) was the number of transcripts, 14,772. The *q*-value was set at 0.05, which led to a threshold of − log_10_(*p*) = 3.7 for the LN treatment and − log_10_(*p*) = 3.9 for the HP treatment. To keep comparisons straightforward (similar effect sizes), analyses were conducted at the most stringent threshold (− log_10_(*p*) > 3.9).

The eQTL types (*cis* or *trans*) were called based on distance to the gene encoding the affected transcript. A *trans*-eQTL had to be located at least 1 Mb from the gene. Furthermore, we calculated the confidence interval of the QTL as a 1.5-drop from the highest − log_10_(*p*). For a *trans*-eQTL to be called, the location of the affect transcript was required to be outside of this confidence interval as well.

### Trans-band identification

Identification of regulatory hotspots (*trans*-bands) was based on assessing whether the number of *trans*-eQTLs mapped to a locus exceeded the expected number based on an equal genome-wide distribution [as in Rockman et al. 2010; Snoek et al. 2017]. We used a Poisson distribution to ascertain the significance of eQTL abundances per 2 Mb bin. For the HP treatment, we expected 15.8 *trans*-eQTL per bin, and for the LN treatment we expected 40.8 *trans*-eQTL per bin. We used a conservative threshold for calling a bin enriched in *trans*-eQTL, *p* < 0.0001. After identifying significant bins, adjacent bins (significant bins, with up to 1 non-significant bin in-between) were merged to a single *trans*-band.

### Enrichment

GO enrichment was determined using the hypergeometric test in R on the GO annotation done for ITAG2.4 downloaded from AgriGO (www.bioinfo.cau.edu.cn/agriGO) (Tian et al. 2017) combined with the annotation for ITAG3.1 and expanded with the GO annotation of the Arabidopsis homologues. All expressed genes were used as background genes in the enrichment test.

### Map and eQTL data in TomQTL

The physical map of the RIL population and the eQTL −log10(*p*-value) scores are available for download and online exploration in TomQTL at http://www.bioinformatics.nl/TomQTL/, an interactive website based on AraQTL (Nijveen et al. 2017) and WormQTL2 (Snoek et al. 2020).

## Supplementary Information

Below is the link to the electronic supplementary material.

Supplementary file1 (DOCX 16 kb)

Supplementary file2 (XLSX 11449 kb)

Supplementary file3 (PDF 75 kb)

Supplementary file4 (PDF 91 kb)

Supplementary file5 (PDF 91 kb)

Supplementary file6 (PDF 91 kb)

Supplementary file7 (PDF 91 kb)

## Data Availability

The datasets generated and analyzed during the current study are available in the Sequence Read Archive (SRA; www.ncbi.nlm.nih.gov/sra) repository, under ID: PRJNA704909.

## Declarations

### One sentence summary

The maternal nutritional environment has a clear influence on the expression of genes in mature dry tomato seeds and the loci affecting this expression shed light on regulation of seed quality.
