# Supplementary material for: Plasticity of maternal environment-dependent expression-QTLs of tomato seeds
Source: Theor Appl Genet. 2023 Feb 22;136(2):28. doi: 10.1007/s00122-023-04322-0 (PMC9944408; doi:10.1007/s00122-023-04322-0)
Supplement: Supplementary file 1 — Supplementary file1 (DOCX 16 kb) [file 122_2023_4322_MOESM1_ESM.docx]

**Supplementary tables and figures legends**

**Supplementary table 1**: **Genetic map of the parental and Recombinant Inbred Lines used**. Matrix of the 101 lines used, 3 F1 heterozygotes (columns) and the 4,515 detected markers listed per chromosome (ch01-ch12) (lines). The genotypes are likelihood based where “0” indicates a locus derived from MM and “1” indicates a locus derived from PI. Chromosome number and genomic position (basepair) are given in the first two columns. Position is the average basepair position of the 100 SNPs sliding bin used to determine the parental origin of the locus.

**Supplementary table 2**: **Introgression size statistics**. Minimum, maximum, mean and median introgression sizes per chromosome (first column).

**Supplementary table 3**: Outcome of a linear model to detect differentially abundant mRNAs between the HP versus LN-treated RILs. For the mRNAs, two identifiers are given in the columns: identifier, and Name. Furthermore, the location (chromosome number and genome position of gene start in basepairs), orientation (+ or – strand), and size (length in basepairs) are indicated. Then, the outcome of the linear model is listed, first the significance in -log_10_(p) followed by two types of corrections for multiple testing: Bonferroni (conservative, as used in the main text) and Benjamini Hochberg False-discovery rate (FDR; less conservative for comparison). The column effect describes the difference between HP and LN treated maternal environment. A description of the effect direction (treatment) is given in the last column.

**Supplementary table 4**: Gene Ontology (GO) enrichment data of maternal environment-related mRNAs. Shown are the GO bin ID, GO bin category (GO name), GO aspect; molecular function (F), cellular component (C), and biological process (P), P value (p.value), Total mRNA’s identified in mRNA bin and GO (in.set), total number of genes in GO (In.GO) and total mRNA set size (set.size).

**Supplementary table 5**: Outcome of a linear model to detect differentially abundant mRNAs between the MM and PI parental lines and their interaction with the environment. For the mRNAs, two identifiers are given in the columns: identifier, and Name. Furthermore, the location (chromosome number and genome position of gene start in basepairs), orientation (+ or – strand), size (length in basepairs) are indicated. Then, the outcome of the linear model is listed, first the tested factor, then significance in -log_10_(p) and a correction for multiple testing (Benjamini Hochberg (FDR)). The column effect describes the difference between the factors tested and the interpretation of the effect direction is given in the last column.

**Supplementary table 6a**: Gene Ontology enrichment analysis of mRNAs that are higher in parental lines MM (left), PI (middle) and their interaction (right). Significantly different genes were taken from the model (see methods and material) only including the parental lines. See legend table S4 for details and abbreviations.

**Supplementary table 6b**: Gene Ontology enrichment analysis of mRNA differences between the parental lines and nutrient environment; higher in HP (left) or higher in LN (right). Significantly different genes were taken from the model (see methods and material) including the parental lines and the nutritional environment. See legend table S4 for details and abbreviations.

**Supplementary table 7a**: Heritability of mRNA abundances from the HP and LN maternal environments. The treatment column indicates the maternal nutrient environment, the mRNA ID is specified in the trait column. H2_keurentjes is the heritability, which was calculated using the genotypic variance (Vg) and the residual variance (Ve) as described in Keurentjes *et al*. (2007) ([Keurentjes et al. 2007](#_ENREF_39)). The FDR column indicates the FDR = 0.05 threshold as determined by 1,000 permutations. The last two columns specify if an mRNA abundance was significantly heritable and whether it was specific for one or multiple maternal environments, or not (group).

**Supplementary table 7b**: Gene Ontology enrichment analysis of mRNAs with significant heritability.

**Supplementary table 8a**: Transgression for mRNA abundances from the HP and LN maternal environments in the RILs. The treatment column indicates the maternal environment, the mRNA ID is specified in the trait column. The n_lines_transgression column specifies how many RILs displayed transgression. The FDR column shows how many RILs showed transgression at the FDR = 0.05 threshold as determined by 1,000 permutations. The last two columns specify if an mRNA abundance was significantly transgressive or not and whether it was specific for one or multiple maternal environments, or not (group).

**Supplementary table 8b**: Gene Ontology enrichment analysis of mRNAs with significant transgression. See legend table S4 for details and abbreviations.

**Supplementary table 9**: Number of eQTLs detected per chromosome and treatment (LN; upper table, HP; lower table). Indicated are for all eQTLs, QTL type (*cis* or *trans*) and QTL effect found per chromosome per nutrient environment (+ or -). The last column indicates the number of eQTLs in the *trans*-bands (TB).

**Supplementary table 10**: List with eQTLs mapped in both the LN and HP maternal nutrient environments. First, the maternal environment is listed, second the mRNA ID (trait). Then columns with the location information of the eQTL: chromosome number, location (bp; and the confidence interval bp_left and bp_right), and the marker. Then, the significance in -log_10_(p) is given and the effect size (negative is higher in MM-derived loci; positive is higher in PI-derived loci). Furthermore, the type of QTL is given (*cis* or *trans*) and whether the QTL is part of a *trans*-band. Also, the variance explained by a single marker model is given (R2_sm). Subsequently, the name and location (chromosome number and start of the gene in basepairs) of the mRNA is listed.

**Supplementary table 11**: Gene Ontology enrichment in genes with an eQTL. See legend table S4 for details and abbreviations.

**Supplementary table 12**: Gene Ontology enrichment in genes with eQTLs mapping to a *trans-*band. See legend table S4 for details and abbreviations.

**Supplementary table 13:** Recombinant Inbred Lines per treatment.

**Supplementary figure 1:** (**A**) Allele frequency (number) of *S. pimpinellifolium* (PI) alleles for each marker across the chromosomes, considering the RILs that were used for the LN treatment. The dashed black line indicates 50% of the genotypes. (**B**) as in (**A**) but for the RILs that underwent the HP treatment.

**Supplementary figure 2**: Venn-diagrams showing **(A)** the overlap between all nutrient treatment-affected mRNA abundances and HP and LN heritable mRNA abundances and (**B**) mRNAs showing transgressive segregation in the HP and LN treatment.

**Supplementary figure 3**: Venn-diagrams showing the overlap between treatment-affected mRNAs, **(A)** *trans*-eQTLs and (**B)** *cis*-eQTL mapped in the HP and LN nutrient environments.
