# Supplementary figures and images for: Plasticity of maternal environment-dependent expression-QTLs of tomato seeds
Source: Theor Appl Genet. 2023 Feb 22;136(2):28. doi: 10.1007/s00122-023-04322-0 (PMC9944408; doi:10.1007/s00122-023-04322-0)

**A**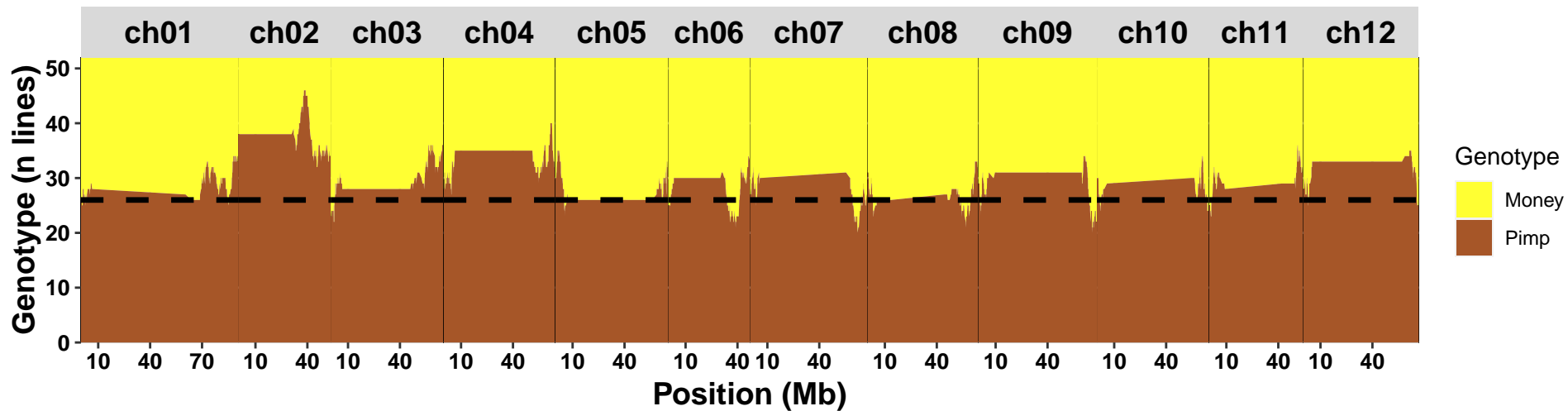**B**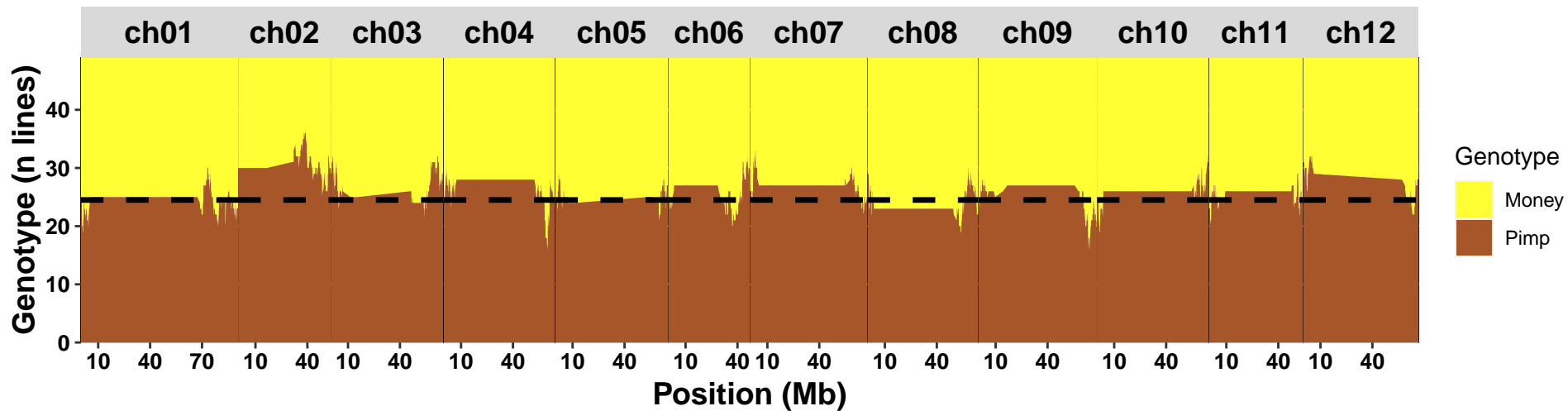

Supplement: Supplementary file 3 — Supplementary file3 (PDF 75 kb) [file 122_2023_4322_MOESM3_ESM.pdf]

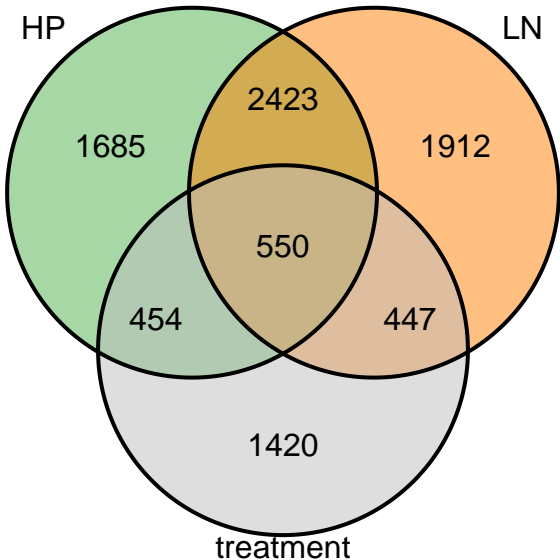

Supplement: Supplementary file 4 — Supplementary file4 (PDF 91 kb) [file 122_2023_4322_MOESM4_ESM.pdf]

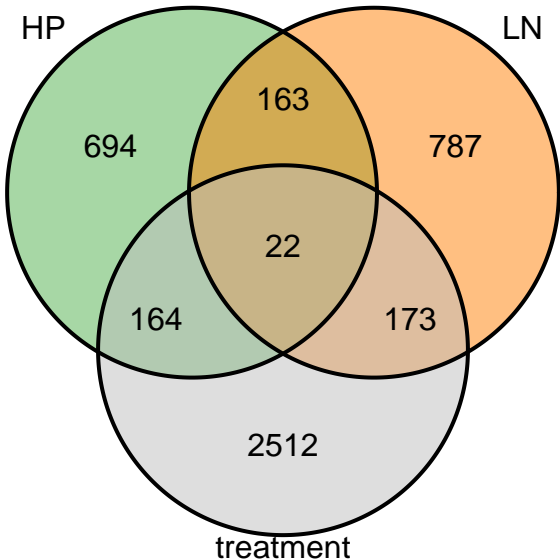

Supplement: Supplementary file 5 — Supplementary file5 (PDF 91 kb) [file 122_2023_4322_MOESM5_ESM.pdf]

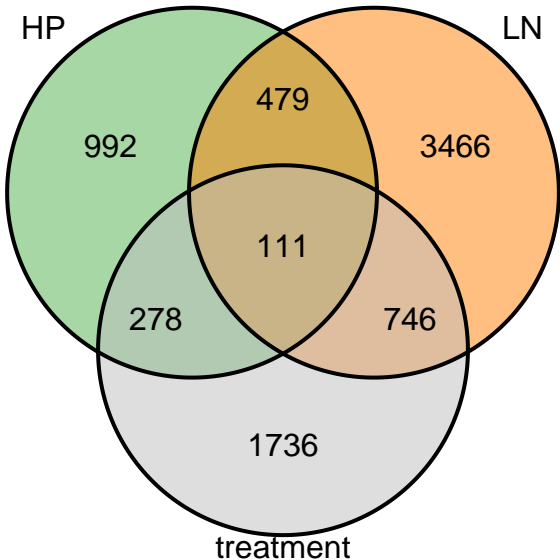

Supplement: Supplementary file 6 — Supplementary file6 (PDF 91 kb) [file 122_2023_4322_MOESM6_ESM.pdf]

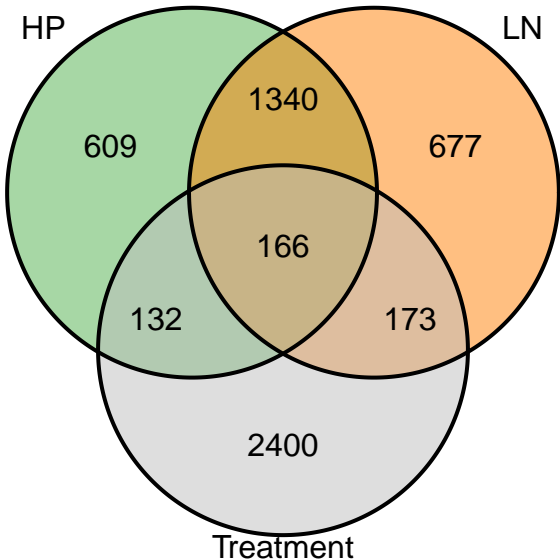

Supplement: Supplementary file 7 — Supplementary file7 (PDF 91 kb) [file 122_2023_4322_MOESM7_ESM.pdf]
